# Labor clauses in trade agreements: Hidden protectionism?

**DOI:** 10.1007/s11558-021-09423-3

**Published:** 2021-05-29

**Authors:** Céline Carrère, Marcelo Olarreaga, Damian Raess

**Affiliations:** 1 Geneva School of Economics and Management, and Global Studies Institute, University of Geneva, Geneva, Switzerland; 2 Geneva School of Economics and Management, University of Geneva, Geneva, Switzerland; 3 World Trade Institute, University of Bern, Bern, Switzerland

**Keywords:** Labor provisions, Preferential trade agreements, Trade flows, Design of international institutions, North–South relations, Gravity equation

## Abstract

We explore the impact of the introduction and design of labor clauses (LCs) in preferential trade agreements (PTAs) on bilateral trade flows over the period 1990–2014. While it is not a priori clear if the inclusion of LCs in PTAs will decrease or increase bilateral trade, we expect the direction of trade to matter, that is, we expect to observe the (negative or positive) impact of LCs in the South-North trade configuration. We also expect, in that configuration, stronger LCs to yield stronger (negative or positive) effects on bilateral trade flows. Using a novel dataset on the content of labor provisions in PTAs, we find in line with our first expectation that while the introduction of LCs has on average no impact on bilateral trade flows, it increases exports of low and middle-income countries with weaker labor standards in North–South trade agreements. Consistent with our second expectation, this positive impact is mostly driven by LCs with institutionalized cooperation provisions. In contrast, LCs with strong enforcement mechanisms do not have a statistically significant impact on exports of developing countries in North–South PTAs. The results are inconsistent with the ideas that LCs are set for protectionist reasons or have protectionist effects, casting doubt on the logic for the reluctance of many developing countries to include LCs in their trade agreements.

**Supplementary Information:**

The online version contains supplementary material (Online Appendix with annexes 1-4) available at 10.1007/s11558-021-09423-3.

In recent decades, developed countries have leveraged their economic and normative power to impose rights-related principles on developing countries as preconditions for access to aid, trade, and investment (e.g., Donno & Neureiter, 2018). Trade-labor linkage strategies are a case in point. The stated objective of labor clauses (LCs) is to protect and promote worker rights among members of preferential trade agreements (PTAs), in line with "fair trade" motives/interests (Ehrlich, 2018), but they are also sometimes seen as a source of trade protectionism. According to Bhagwati (1995), "fair trade" demands for imposing higher labor (and environmental) standards as a condition for market access mainly derive from the desire of labor unions and import-competing producers in high-income countries to protect jobs and profits by shielding industries from increased competition from low-income countries. In this view, invoking "unfairness” of trade due to weak standards in developing countries and related demands to "level the playing field" are aimed at reducing trade pressure and thus getting protection. Drawing on this argument, developing countries have historically adopted a defensive stance towards the inclusion of LCs in PTAs (e.g., da Motta Veiga & Rios, 2008). International political economy scholarship lends credence to the claim that protectionist concerns in high-income countries play a significant role in the rise and governance of fair trade standards in PTAs (Hafner-Burton, 2009; Lechner, 2016; Postnikov & Bastiaens, 2020; see also Bastiaens & Postnikov, 2020; and Hafner-Burton et al., 2019).

If protectionism drives trade-labor linkages, one would expect LCs to act as hidden trade barriers and, accordingly, to be associated with reduced trade. This paper tests this proposition by examining the impact that the introduction of LCs as well as various types of LCs in PTAs has on bilateral trade flows. To measure the trade effects that LCs have we use a new dataset that provides detailed information on the inclusion and design of labor provisions in PTAs since the early 1990s. Labor provisions in PTAs vary considerably in terms of their scope and stringency. At the one end of the spectrum, we find shallow LCs with a single reference to "improve working conditions" in the preamble (e.g., Chile-Ecuador PTA of 2008). At the other end, we find LCs with stringent provisions, such as those PTAs with substantive commitments to improve labor standards in the main text that are legally binding and strongly enforceable (e.g., US-Jordan PTA of 2000), those with strongly institutionalized cooperation mechanisms over labor-related commitments (e.g., EC-Korea PTA of 2010), or those including both these features (e.g., Canada-Colombia PTA of 2008).

Despite widespread beliefs that LCs amount to disguised protectionism, it is not a priori clear whether the inclusion of LCs in PTAs will actually reduce or increase trade. Whether the motives for the introduction of LCs are sincere (as in fair trade) or not, the effects of LCs can be protectionist. If LCs improve labor practices and then labor costs in developing countries, it can lead to a deterioration in their comparative or competitive advantage and thus to reduced trade. Moreover, LCs in PTAs can lead to the deterioration of market access for low-income countries in breach of labor rights requirements in PTAs if advanced economies use enforceable LCs to withdraw trade concessions.

Others have argued that the external enforcement of minimum labor standards via LCs can help increase the demand for products from the South in the North, whether or not LCs affect labor conditions, leading to more trade. First, LCs can signal to concerned consumers and firms in the North that there is adequate worker protection in the low-income partner. Second, strong LCs can be in the interest of low-income countries that lack the capacity to implement their own labor laws as they can help enforce existing laws and reduce the uncertainty associated with their enforcement. Given that developed countries have increasingly placed value on strong labor protection, raising standards can result in developing countries becoming more competitive in developed markets. Polaski (2003) offers an interesting discussion of how the LC in the US-Cambodia PTA helped the Cambodian government implement worker protection policies, which the government considered desirable, but would have been impossible to implement without the PTA given the political economy at the time in Cambodia. Moreover, LCs may be linked to more trade by way of a supply-side mechanism running through improvements in labor standards and productivity (Brown et al., 2013).

The direction of trade matters for both arguments as they focus on the impact of LCs on exports from developing countries with weaker labor standards to developed countries with stronger standards and concerned consumers and firms. Accordingly, we expect LCs to increase or decrease bilateral trade flows when the exporter is a developing country with weaker labor protection and the importer a developed country with stronger labor protection. We not only consider heterogeneous effects across different trading partners (North–North, South-South, and North–South PTAs), but also across pairs of trading countries with differences in worker protection. In addition, because they send a stronger signal about sufficient worker protections in partner countries and/or because they are more likely to pull up working conditions in developing countries, we also expect strong LCs to have a stronger impact (positive or negative) on exports of low or middle-income countries to high income countries. This includes LCs with strongly enforceable standards and LCs with strongly institutionalized cooperation mechanisms.

Using a gravity model, our empirical analysis suggests that on average the inclusion of LCs does not have a statistically significant impact on bilateral trade flows. However, exports of low and middle-income countries benefit from the inclusion of LCs in North–South PTAs. Consistent with this finding, we show that when the exporter has weaker labor rights than the importer, the impact of the introduction of LCs in PTAs on trade flows is positive. Moreover, the positive effect on exports of low and middle-income countries within North–South PTAs is mainly driven by LCs with institutionalized cooperation mechanisms. LCs with strong enforcement mechanisms, in contrast, do not have a statistically significant impact on the exports of low and middle-income countries within North–South PTAs, even when they are associated with institutionalized cooperation. Our empirical strategy addresses in various ways endogeneity concerns that plague studies of the effect of treaty provisions (e.g., Hill, 2010; Spilker & Böhmelt, 2013).

Our paper contributes to the literature on the effect of the design of PTAs on trade flows (for a recent review of that literature, see Baccini, 2019). First, the number of LCs in PTAs has expanded rapidly over the past three decades: 27 percent of PTAs signed in 1995 included LCs, 26 percent in 2000, 44 percent in 2005, 67 percent in 2010 and 71 percent in 2014. Thus, understanding the consequences of this evolution in the design of PTAs is important. Specifically, understanding whether the inclusion of LCs in PTAs reduces or increases trade from low-income countries is an open question: is the reduction in the comparative advantage of low-income countries associated with stronger worker protection (and the possibility of protectionism in the North) larger than the increase in demand in the North for goods produced with higher labor standards? In both academic and policy circles this question has long been debated on the basis of pre-existing beliefs rather than sound empirical evidence. As surprising as it may sound, this is a million-dollar question still awaiting a convincing answer.^1^

Second, to design better and more efficient PTAs containing LCs, we need to understand how different provisions contained in LCs, such as enforcement and cooperation, affect trade flows. While strong LCs may improve labor standards, they may also lead to an increase or fall in trade flows among trading partners. Data constraints on the design of labor provisions in PTAs has until now prevented scholars from considering this aspect.

Our paper also contributes to academic debates about whether preferential trade liberalization facilitates or undermines multilateral trade liberalization. A better understanding of the effects of LCs in PTAs can inform the desirability regarding the inclusion of a labor agreement in multilateral negotiations. The unsuccessful International Trade Organization that resulted in the GATT in 1948 was expected to include labor rights commitments. This was excluded from the GATT apart from article XX(e), which allows to withdraw market access concessions if the goods were produced using prison labor. At the creation of the World Trade Organization (WTO) in 1994, developing countries opposed the introduction of a LC. The issue reappeared during the Singapore Ministerial Conference of 1996, and then later buried in the Cancun Ministerial of the Doha Round. Now with the Doha Round out of the way, the WTO is looking for new avenues for countries to engage at the multilateral level. A labor agreement in the WTO can be feasible and desirable if it leads to more, not less, trade.

## Trade-labor linkages

In the post-World War II period, state-led linkages between trade and worker rights emerged in the early 1990s.^2^ Frontrunners were African nations that established the African Economic Community in 1991 and the European Community (EC) with its trade agreements signed with Hungary and Poland in 1991 (Raess & Sari, 2018, 2020a).^3^ The former PTA foresees the enactment of measures in order to protect the child against abuse and exploitation, cooperation over labor laws, and the setting-up a committee on Health, Labour and Social Affairs. The latter PTAs, which are identical with regard to labor provisions, include a substantive commitment to conditions of work, its enforcement through dispute settlement with the possibility to unilaterally retaliate (though coming short of monetary and trade sanctions), and cooperation over workplace health and safety taking as a reference the level of protection in the EC. The EC negotiated many such agreements with the Central and Eastern European (CEE) countries in the early/mid 1990s.

Against this backdrop, the depiction of the North American Free Trade Agreement (NAFTA), signed in 1992 between Canada, Mexico and the US, by President Clinton in 1993 as the first agreement that ever really got "any teeth in what another country had to do with its own workers and its own labor standards" (Broken Promises: 1) was at the time (only partly) accurate. NAFTA’s side agreement on labor, the North American Agreement on Labor Cooperation (NAALC), includes legally binding commitments with respect to the fundamental workers’ rights, conditions of work as pertains to health and safety and wages, as well as the effective enforcement of domestic laws. The latter is enforceable through binding state-to-state dispute settlement with possible retaliation measures including trade sanctions, monetary compensation, and other measures deemed appropriate. It also has an extensive list of issues and means for cooperation and capacity building activities, and it is the first PTA to set up a separate body with regular meetings in charge of implementing the labor provisions. The only other comprehensive LC during this period is found in the Canada-Chile PTA of 1996, a LC that was molded on NAALC and is virtually identical to it.

With the failed attempt to introduce a LC at the Singapore Conference of the WTO in 1996, due to the opposition of developing country governments, the US and the EU (and Canada) started pushing the social agenda in PTAs through the bilateral channel. By 2016, the US had 14 PTAs with 20 countries (Broken Promises: 2).^4^ US PTAs contain far-reaching and strongly enforceable labor provisions. With one exception (the US-Jordan PTA of 2000), they also contain strongly institutionalized cooperation mechanisms. The first EU agreement with references to the ILO’s four fundamental workers’ rights^5^ was the PTA signed with South Africa in 1999. Relatively broad in terms of labor-related cooperation provisions but with a weak institutional framework, this agreement does not entail enforceable labor standards. Many subsequent EU PTAs do not come with strong labor-related enforcement mechanisms. While it is common to portray US PTAs as enforceable and EU PTAs as not enforceable with respect to labor standards (e.g., Postnikov & Bastiaens, 2014), such a depiction does not do justice to the evolution of the design of LCs in EU PTAs, as we have already seen with the earlier generation of EC agreements with CEE countries. To illustrate with a recent example, in the CARIFORUM-EC Economic Partnership Agreement of 2008, substantive commitments over fundamental worker rights and commitments not to derogate from existing labor laws are strongly enforceable through dispute settlement. Indeed, Art. 213 allows for unilateral "*appropriate measures*", other than the suspension of trade concessions, to be adopted by the complaining party.

## Literature review

Due to the relative novelty of the growth of LCs in PTAs and the lack of available data, this area of research has until recently been characterized by a dearth of quantitative studies on the impact of LCs in PTAs. Most impact studies have focused on the effect of LCs on labor rights and conditions. Kim (2012) shows that labor provisions in PTAs of a large developed economy lead to improvements in labor rights in partner countries prior to signing the agreements, arguing that these countries act upon the belief that upgrading labor standards will increase their attractiveness as trade partners as well as their competitiveness in developed economies. This study only considers 12 US PTAs signed between 1985 and 2006. Focusing on the EU trade-labor linkage approach which stresses "soft" mechanisms such as dialogue and capacity building, Postnikov and Bastiaens (2014) find that EU PTAs with labor provisions correlate with increased compliance with labor rights in partner states, an effect that is exhibited ex post during the implementation phase. The sample consists of 18 EU PTAs ratified between 1995 and 2010. Both studies are relatively narrow in their scope as they focus on only one major player. Using a global sample of PTAs for the period 1990–2015, a working paper by Raess and Sari (2020b) finds that only membership in PTAs with a certain type of LCs, namely institutionalized cooperation mechanisms, reduces labor rights violations among a large sample of developing countries. Other statistical studies using a large-N sample of PTAs tend to find no impact on working conditions (ILO, 2016; Kamata, 2016, 2018).

Economic studies have focused on the impact of differing *domestic* labor standards on trade flows, with inconclusive results, mostly insignificant (see Aggarwal, 1995; Brown, 2000; Rodrik, 1996; but see Busse, 2002). Economists and political scientists have looked at the trade effect of different kinds of PTAs in terms of depth of the agreements. They find that the deeper is the PTA, the stronger is the impact on trade among members (e.g., Baier & Bergstrand, 2007; Cipollina & Salvatici, 2010; Dür et al., 2014; Vicard, 2009).

To our knowledge, no study has examined the impact of LCs on trade flows until ILO (2016).^6^ Based on an analysis of 260 trade agreements notified to the WTO for the period 1995–2014, the ILO study finds that on average both PTAs with and without LCs significantly increase bilateral trade flows and that the two effects are not statistically different from one another. In addition, it finds that PTAs with LCs have a significantly stronger positive impact on bilateral trade flows in South-South PTAs and on exports from high-income countries in North–South PTAs. These results are difficult to square with theoretical expectations. Note that the study does not provide results for the effect of PTAs with different types of LCs.

The literature on the determinants of, and the motivations for, the inclusion of LCs in PTAs has weighed in on the issue of whether LCs reflect protectionist interests. Lechner (2016) finds that strong import pressure and a high wage differential between the trade parties are associated with more stringent social clauses in PTAs while Raess et al. (2018) show that a country’s skill profile measured as the strength of skilled to unskilled workers is negatively correlated with the depth of labor provisions in PTAs, suggesting that protectionist concerns play a role in issue linkage. Postnikov and Bastiaens (2020) argue and show that Northern countries with majoritarian electoral systems have strongly enforceable social standards in their PTAs whereas those with proportional systems have not, indicating that the former exhibit a social protectionist bias because majoritarian systems are more conducive to the mobilization of protectionist interests. While trade unions in advanced economies are often seen as one of the interest groups that may use LCs in PTAs to placate competitive pressures to protect jobs and wages, they are also global-norm advocates that seek to spread international principles and rights at work. Powerful trade unions have strongly shaped the inclusion and design of labor provisions in PTAs (Hafner-Burton, 2009; Raess et al., 2018), which may or may not point to protectionist concerns. Finally, public opinion research on social standards in PTAs and fair trade consumption in advanced economies finds that support for such policies and purchasing behavior are at least partially motivated by sincere moral concerns about poor labor and environmental conditions abroad (Bastiaens & Postnikov, 2020; Ehrlich, 2018). Overall, while the evidence is mixed, existing studies lend some support for the view that protectionist concerns play a role in the politics of linking trade-labor issues. Do (partly) protectionist motives translate into protectionist effects?

## The argument

There are several dimensions behind predicting and understanding if and how labor clauses affect trading partner’s trade. The first is if and how LCs affect labor standards and then how does that impact trade. The second dimension is if the relationship between LCs and trade differs across different trading partners. The third dimension is if the trade effect varies across types of LCs.

A body of literature bemoans the imposition of developed country labor standards on developing country exporters by way of the inclusion of LCs in PTAs. The rallying cry for developing countries, supported by orthodox economists, has been that LCs lead to a deterioration of their comparative advantage (Bhagwati, 1995). The assumptions underlying this claim are twofold: labor standards negatively correlate with international trade; and that LCs affect labor standards. Regarding the former, developing countries tend to be abundant in unskilled labor (and natural resources) and scarce in capital. Their competitiveness lies in cheap labor (and abundant resources). These countries thus derive comparative advantage (and export performance) in unskilled-labor-intensive goods from weak labor standards (Busse, 2002). The impact of higher labor standards is likely to be felt most strongly in unskilled-labor-intensive production, since improvements in working conditions increase labor costs which in turn reduce production and exports of labor-intensive goods. In other words, stronger worker protection in developing countries can result in a deterioration of their comparative advantage (Golub, 1997; Panagariya, 2006). Regarding the latter, research has shown that LCs can under certain conditions improve labor standards in developing countries (ILO, 2013; Kim, 2012; Postnikov & Bastiaens, 2014).

Higher labor standards, however, are not necessarily inimical to firms’ international competitiveness so that LCs, via its effect on labor conditions, can actually lead to more trade. Treating workers well, for instance by paying them generously or providing safe working environments, institutional mechanisms for worker voice and on-the-job training, may enhance worker motivation which in turn may increase labor productivity and firm competitiveness, including export performance. Researchers have found this supply-side mechanism to operate in institutions that condition the effect of trade on labor standards in developing countries. In the Dominican Republic (DR) under the DR-Central America PTA of 2004, Schrank (2013) found that trade-backed labor standards conditionality helped tackle rights violations via more labor inspections and fostered skill-building, which he interprets as evidence that social protection/investment can be reconciled with strong firm performance. Using data from Better Factories Cambodia,^7^ Brown et al. (2013) find that compliance with labor standards is positively associated with firm survival. Compliance allegedly induced firms to experiment with more humane labor-management practices that led to greater efficiency.

There is also a "demand-side" explanation underpinning the logic of LCs leading to more trade. LCs can help firms in the South signal to consumers and firms in the North about (adequate) conditions of work, thereby increasing the demand for goods produced by firms located in countries that have signed PTAs with LCs. This is an argument about the role of the socially conscious consumers and firms engaged in global supply chains in the North. When blatant labor rights violations in the global factories made the headlines in the 1990s, activist groups such as the anti-sweatshop movement began to thematize exploitative working conditions in global production networks and target large corporations such as Nike. Under increased media scrutiny and concerned about their reputation, lead firms in the North set up voluntary codes of conducts destined to their suppliers to address poor labor standards in their supply chains (Bartley, 2018; Locke, 2013). Firms’ response was accompanied, if not propelled, by increased consumer demand for goods produced under decent conditions of work (Elliott & Freeman, 2003; Hainmueller & Hiscox, 2015).^8^ Concerned groups in the North about adequate standards abroad in the context of trade can be subsumed under the category of fair traders (Ehrlich, 2018). They express their concern both through private action (e.g., boycott of products made with child labor, purchase of products labelled "fair trade") and through government regulation. A manifestation of the latter is the attempt to regulate trade through the inclusion of labor, environmental and human rights provisions in PTAs. It has been estimated that fair traders make up as much as a third of the population in developed countries (Ehrlich, 2018).

Research suggests that concerned consumers and firms in the North, or fair trade interests, can have an impact on both labor conditions and international trade. Empirical evidence suggests that labor standards in developing countries improve when they trade with countries that have strong labor protections and activist groups (Greenhill et al., 2009). Moreover, surveys of Vietnamese exporters suggest that firms are more willing to invest in improving labor standards when they have the opportunity to access export markets that have importers concerned about labor practices (Malesky & Mosley, 2018). Empirical evidence also suggests that (information about) compliance with labor and environmental standards can positively affect firm-level trade in the developing world. Drawing on financial transactions of several thousand firms that export through a global sourcing agent, Distelhorst and Locke (2018) find that compliance with social standards by developing country manufacturing firms can lead to modest increases in annual purchasing by importers in developed countries.^9^ In addition, the literature on micro-level preferences for social standards in PTAs find that individuals in developed countries more strongly support free trade when PTAs include labor and environmental standards (Bastiaens & Postnikov, 2020), evidence found to be consistent with an ex-ante reassurance mechanism and, especially, the fair trade norms hypothesis.

Even if LCs are ineffective at improving labor standards, they can change bilateral trade flows. Again, there are valid theoretical arguments on both sides of the debate as to whether LCs in PTAs result in more or less trade. On the one hand, if LCs respond to protectionist (rather than fair trade) interests in the North, developed countries might use them to curtail preferential market access of exporting countries in breach of their obligations in relation to labor rights (Bhagwati, 1995). This can occur when PTAs include binding commitments over labor standards that are enforceable through dispute settlement. A party to such an agreement may refer a complaint against a non-complying trade partner to a panel of experts for arbitration. If the panel rules against the party complained against, the complaining party may be entitled to take appropriate retaliation measures, such as the imposition of trade sanctions, specifically the withdrawal of the benefits of better market access.

On the other hand, concerned consumers and firms in the North might use LCs as a cognitive shortcut for adequate labor standards in trading partners irrespective of the actual level of labor protection in developing countries or improvements induced by the LCs, information that in turn might influence their purchasing decisions. In other words, the Northern public might not need to see change in labor practices if the signal provided by LCs is powerful enough.

Regardless of whether LCs lead to more or less trade, all the above arguments, including protectionist and fair trade mechanisms, focus on the impact on exports from low-income countries with weaker labor standards to high-income countries with stronger standards and more concerned consumers and firms. Thus, the direction of trade matters. Therefore, we expect to observe the (positive or negative) impact of LCs when the exporter is a low or middle-income country and the importer is a high-income country.

It is conceivable that the two theories could both be at work. For instance, LCs might deteriorate comparative advantage in developing countries by increasing labor costs, yet consumers may respond positively to the signal of the LCs thereby increasing demand. Since the two push in opposite directions on the volume of trade, we may not observe a difference between South-North trade and other configurations (i.e., the situations where the exporter and the importer are both low or middle-income countries, both high-income countries, or where the exporter is a high-income country and the importer a low or middle-income country). If so, we do not know whether both or neither phenomena are at work. In the empirical test, the sign of the coefficient for LCs in the reduced sample in which the exporter is a low or middle-income country and the importer is a high-income country will tell us which phenomenon is more important for understanding the impact of LCs on trade. Hence our first two hypotheses:

- *Hypothesis 1a*: LCs in PTAs decrease exports of low or middle-income countries with weaker labor standards to high-income countries with stronger labor standards.
- *Hypothesis 1b*: LCs in PTAs increase exports of low or middle-income countries with weaker labor standards to high-income countries with stronger labor standards.

We also expect stronger LCs to have a stronger impact (positive or negative) in North–South agreements compared to weaker LCs because stronger enforcement and institutionalization mechanisms are more likely to lead to labor upgrading and because of higher reliability of the signal. Shallow labor provisions not supported by enforcement capacity, either in the form of dispute settlement or capacity building mechanisms, are unlikely to improve labor conditions and thus to provide a credible signal. Indeed, statistical studies on the impact of LCs on working conditions using a dummy variable for LCs tend to find no effect (e.g., ILO, 2016; Kamata, 2016, 2018; Posso, 2017).

By contrast, stronger LCs are more likely to improve labor standards. Two types of LCs stand out. First, enforceable LCs through sanctions may entail strong material incentives to comply with labor provisions in PTAs as failure to do so can jeopardize market access (Hafner-Burton, 2005). This should hold in particular for developing countries exporting to developed countries in the North that tend to have large markets. Enforceable LCs through dispute settlement backed by sanctions may also send a stronger signal to concerned Northern consumers and firms about strong labor-related commitments and better labor standards in the trade partner countries.

Second, the external enforcement of labor standards through LCs with strong mechanisms of institutionalized cooperation includes mechanisms such as dialogue, technical assistance and capacity building activities, taking place in the context of a strong institutional framework characterized by a specialized body in charge of the monitoring and implementation of the labor provisions (e.g., Labor Affairs Council) and the involvement of civil society organizations (social partners, NGOs) and/or the ILO.^10^ LCs that emphasize strong and inclusive intergovernmental institutions dedicated to labor cooperation provide a venue where trade partners can regularly meet and exchange information and thus have the possibility to socialize with each other and develop identities (Pevehouse, 2005). States that primarily view strong worker protection as a liability may learn about inalienable labor rights and productivity gains associated with an efficiency wage and compliance with fundamental labor rights more generally. In addition, in North–South PTAs, LCs that emphasize capacity building, whereby developed countries provide much needed financial and organizational resources (e.g., expertise in labor law, training of labor inspectors), are more likely to help developing countries improve labor standards and realize productivity gains. These type of LCs may thus equally act as a signal to consumers and firms in the North about effective enforcement capacity to address poor labor standards in developing countries.

Some empirical studies suggest that strong LCs can improve labor standards in the developing world. However, the timing of the effect appears to vary across the two types of LCs, with the sanction model displaying an ex ante effect (ILO, 2016; Kim, 2012) while the institutionalized cooperation model has an ex post effect (Postnikov & Bastiaens, 2014; Raess & Sari, 2020b).^11^

To sum up, just like LCs, strong LCs can affect labor outcomes which in turn can impact trade either positively (via the supply- and/or demand-side mechanisms) or negatively (via deterioration of comparative advantage). However, they are more likely to do so than LCs with low stringency. In the absence of an effect on labor standards, strong LCs might still affect trade more strongly than weak LCs because they arguably provide a more credible signal. Moreover, in the scenario where LCs are motivated by protectionist interests, strong LCs with enforcement mechanisms backed up by sanctions are a necessary condition for developed countries with stronger labor standards to use LCs to withdraw preferential market access. All in all, we have thus our next two hypotheses:

- *Hypothesis 2a*: The (positive or negative) impact of LCs in PTAs on bilateral trade flows when the exporter is a low or middle-income country with weaker labor standards and the importer a high-income country with stronger labor standards is stronger when the LCs are accompanied with strong enforcement mechanisms.
- *Hypothesis 2b*: The (positive or negative) impact of LCs in PTAs on bilateral trade flows when the exporter is a low or middle-income country with weaker labor standards and the importer a high-income country with stronger labor standards is stronger when the LCs are accompanied by strongly institutionalized cooperation mechanisms.

## Labor clauses in PTAs: data and descriptive statistics

A major challenge was to collect information on the existence and design of LCs for a large sample of PTAs with a global scope. Until recently there was no such dataset available. Existing datasets on labor provisions, in most cases created as part of a broader mapping exercise of nontrade-issues in PTAs, provide relatively crude measures of the content and stringency of labor commitments that are inadequate for the purpose of this paper (Hofmann et al., 2019; Kamata, 2016; Lechner, 2016; Milewicz et al., 2018).^12^ We use a new dataset that systematically documents labor provisions in PTAs (LABPTA dataset; for details, see Raess & Sari, 2018). The LABPTA dataset contains 487 preferential PTAs for the period 1990–2015 that come from the DESTA dataset, the most comprehensive dataset in terms of the number of PTAs covered (Dür et al., 2014). PTAs are agreements liberalizing trade between two or more countries without extending this liberalization to all countries.

A PTA is coded as having a LC if it has at least one provision in the preamble or in the main text meant to protect and/or promote worker rights and conditions of work.^13^ In terms of specific design features of LCs, we focus on two dimensions of the stringency of labor provisions, namely strong enforcement and institutionalized cooperation (see Raess & Sari, 2018). These amount to different tools for the effective implementation of labor provisions. The former captures so-called “hard” labor rights standards (Hafner-Burton, 2005; Postnikov & Bastiaens, 2014). Specifically, a LC is coded as having strong enforcement mechanisms if a PTA (i) has at least one substantive labor provision covered by either quasi-judicial (e.g., third-party adjudication) or judicial (e.g., standing judicial court) dispute settlement *and* (ii) allows for the unilateral imposition of sanction measures of at least one of the three following types, "monetary compensation", "trade sanctions" or "other appropriate measures". A LC with strongly institutionalized (or "deep") cooperation mechanisms refers to a PTA with the following two features: (i) at least one substantive labor provision covered by cooperation activities (e.g., technical assistance, capacity building); and (ii) a comprehensive institutional framework establishing a specialized body for the monitoring and implementation of labor provisions *and* allowing for the participation of third parties (social partners, NGOs, ILO, other third parties).

Using the above two types of strong LCs, we decompose the LC variable into four mutually exclusive dummy variables as follows: LC^weak^, LC^enf^, LC^coop^, and LC^enfcoop^. They indicate respectively whether worker rights are mentioned in the PTA without any strong enforcement or institutionalized cooperation (so this corresponds to LCs with low regulatory stringency or weak LCs), whether there is strong enforcement provisions but with no deep cooperation (strong enforcement only), whether there is deep cooperation provisions but with no strong enforcement (deep cooperation only) or whether there is both strong enforcement and deep cooperation provisions (for all variables 1 = yes; 0 = no).

**Fig. 1:**
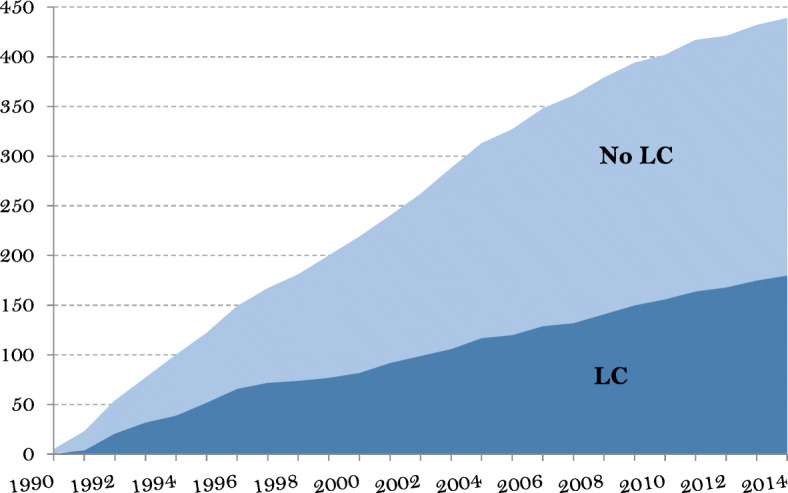
Cumulated number of PTAs signed after 1990 with and without a LC, 1990–2014. Source: authors’ computation

**Fig. 2:**
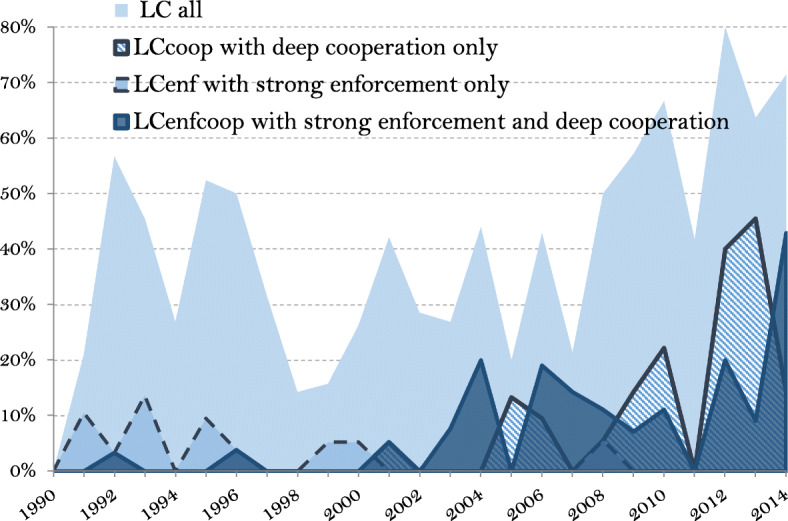
Share of LCs in total PTAs signed after 1990 per year, 1990–2014. Source: authors’ computation

Over the 487 coded PTAs signed or revised after 1990, 437 are included in our sample.^14^ Figure 1 reports the cumulated number of the signed PTA included in the sample over 1990–2014, decomposing between PTAs with and without LCs (potential changes in the LC content of a given agreement over the period are taken into account in the Figure). In 2014, 39 percent of PTAs signed after 1990 included LCs (169 out of 437). Figure 2 shows that more than half of new (or revised) PTAs since 2008 include LCs and mostly with strong enforcement mechanisms and/or deep cooperation designed in the agreement.

**Fig. 3:**
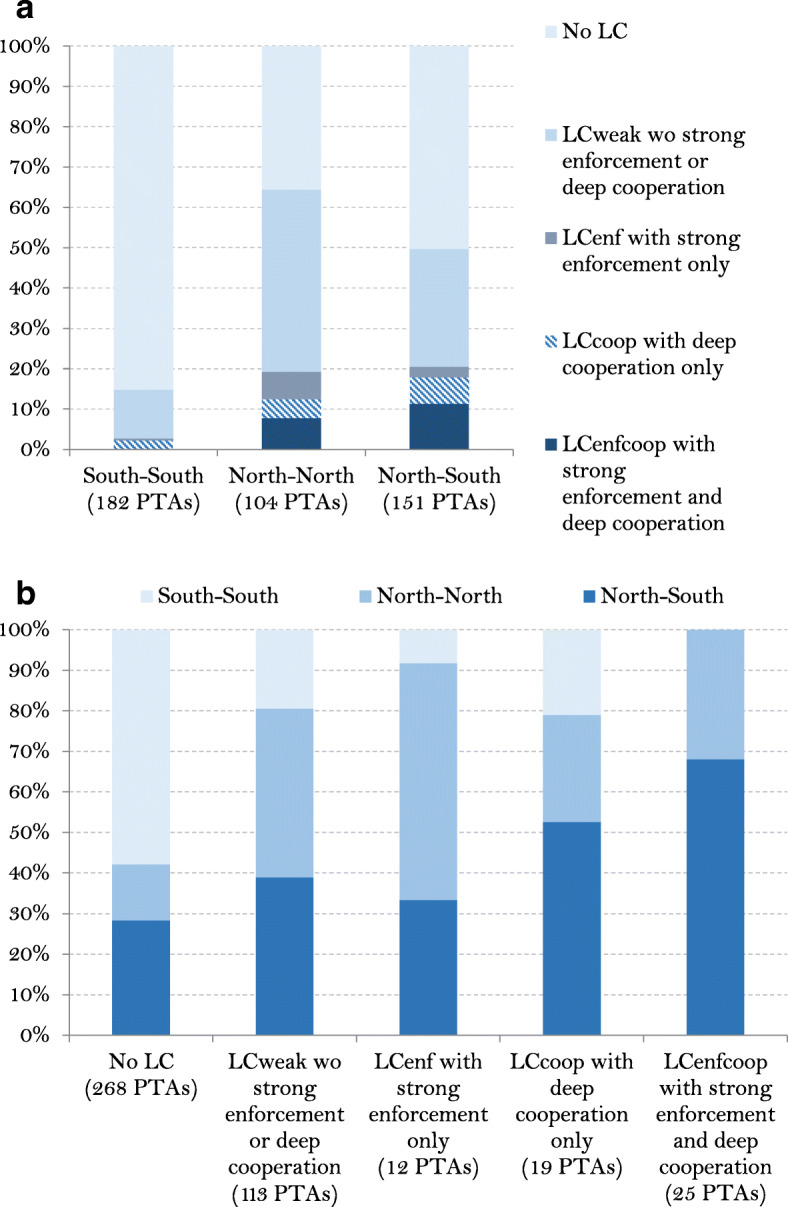
Distribution of PTAs by the level of development of its members and the LC type (cumulated over 1990–2014). a. by level of development. b. by type of LC. Source: authors' computation

Next, we decompose PTAs according to the level of development of its members. We distinguish between North–North, South-South and North–South agreements.^15^ Figure 3a shows that the highest share of LCs is found in North–North agreements (around 64 percent of total North–North PTAs). In 85 percent of South-South agreements, worker rights are not taken into account. Figure 3b confirms these stylized facts. In the subsample of PTAs in which the LC is specified with strong enforcement "only" (12 PTAs), 33 percent implies North–South partners, 58 percent North–North and only 9 percent South-South. In the subsample in which the LC is specified with deep cooperation "only" (19 PTAs), 53 percent implies North–South partners, 26 percent North–North and 21 percent South-South. We do not have any South-South PTAs with LCs including both strong enforcement and deep cooperation in our sample (25 PTAs), these are mainly found in North–South agreements (68 percent).

## Empirical methodology

We follow a standard gravity equation approach to assess the extent to which the inclusion of LCs in PTAs leads to more or less trade (Head and Mayer 2014):

1 $$ \ln {m}_{ij t}={\alpha}_{it}+{\alpha}_{jt}+{X}_{ij}\gamma +\beta {LC}_{ij t}+{\varepsilon}_{ij t} $$

where ln *m*_*ijt*_ is the log of imports of manufacture of country *i* from country *j* at time *t*; *X*_*ij*_ is a vector of usual gravity time invariant determinants of bilateral trade flows that we detail below; *LC*_*ijt*_ is a dummy variable that indicates whether the PTA between countries *i* and *j* in year *t* contains a LC. *α*_*it*_ and *α*_*jt*_ are importing country *i* times year fixed effects and exporting country *j* times year fixed effects. They control for the economic size of the importer, the business cycle, the overall level of protectionism, or the functioning of labor institutions, but also the multilateral resistance terms, which capture the trade frictions between the importer or the exporter and the rest of the world (Anderson and van Wincoop 2003).^16^*ϵ*_*ijt*_ is an independent and identically distributed error term.

Classic gravity variables included in the *X*_*ij*_ matrix are: log(*Distance*_*ij*_), the log of the bilateral distance between the importer *i* and the exporter *j*; *Contiguity*_*ij*_ a dummy variable that indicates whether the two trading partner share a common border; *Common Language*_*ij*_ a dummy variable that indicates whether the two countries share a common language; *Past Colonial*_*ij*_ a dummy variable that indicates whether they share a colonial link; and *Common Colonial*_*ij*_ indicating whether they had a common colonizer. In addition, deeper PTAs tend to create more trade, and they are also probably correlated with the presence of LCs. Therefore, controlling for the depth of PTAs is crucial because we do not want the LC variable to capture the presence of a deeper agreement and more cooperation in general.^17^ We measure the depth of PTAs using a dummy variable where the cutoff is the median of an updated index of PTA depth (from 0 to 7) by Dür et al. (2014). It turns out that the median of *Depth*_*ij*_ indicates whether the agreement has provisions that go beyond market access. Because Dür et al.’s (2014) measure of PTAs depth does not consider labor provisions, it does not already capture the impact of LCs.^18^

Our sample only contains countries that have signed effective PTAs (we have excluded from the sample the few PTAs with PTA depth index of zero). This explains why there is no control for the presence of a PTA in Eq. (1). Our sample only includes bilateral pairs that signed a PTA and we compare those with a LC to those without. This should address concerns regarding reverse causality going from higher trade towards the signing of PTAs. Another interesting aspect is that by focusing only on countries that have PTAs in place, we circumvent the problem of many zero trade flows that may have led to biased results (Santos Silva and Tenreyro 2006).^19^

The coefficient *β* is our parameter of interest and the percentage change in imports of country *i* from country *j* associated with the LC is given by: *e*^*β*^ − 1. Thus, if *β* > 0, the LC increases trade, whereas when *β* < 0, the LC reduces trade flows (using agreements without a LC as the reference group).

To start with, we explore the heterogeneous effect that LCs may have across pairs of countries.^20^ Indeed, the presence of a LC in a PTA among high-income countries with quite protective labor laws should not be expected to have a large impact. In contrast, a PTA with a LC between high and low-income countries with big differences in their labor laws is likely to have a much larger impact (positive or negative) on export flows from the low to the high-income countries. To check for these heterogeneities, we carry out two empirical tests. First, assuming that the level of economic development and the level of worker protection is correlated, we will split the sample into four types of bilateral relationships: a sample containing trade flows among low and middle-income countries (South), a sample containing trade flows among high-income countries (North), a sample containing trade flows from high to low and middle-income countries (North to South), and finally a sample containing trade flows from low and middle-income to high-income countries (South to North).^21^ We expect the coefficient *β* to be significantly different from zero (either positive or negative) for the sample containing export flows from low and middle-income to high-income countries.

Second, because the level of economic development may be imperfectly correlated with worker protection, we will test the heterogeneity of the impact of LCs on trade flows using a direct measure of differences in worker protection across trading partners. Our a priori belief is that the impact (positive or negative) should be found on export flows from countries with weak worker protection to countries with strong worker protection.^22^ As a measure of worker protection, we use the indicator developed by Kucera and Sari (2019), called Labor Rights Indicator (LRI), which captures both de jure and de facto violations of worker’s rights in a scale between 0 and 10 (a larger value means lower worker protection).^23^ For each country pair we then compute the median difference in violations of workers’ rights over the period 1995–2014 between the exporter and the importer. We then construct two variables. One where the differences among trading partners are replaced by zero when the difference is negative (the exporter has less violations of workers’ rights than the importer), which keeps only positive difference and is noted LRI+. The other variable is the mirror image where the differences are replaced by zero when the difference is positive (the exporter has more violations of workers’ rights than the importer), and is noted LRI-. The rationale is that the impact of LCs is expected to be observed when the exporter has weaker worker protection than the importer. We then introduce these two variables as explanatory variables in our main specification in Eq. (1) and interact them with the LC variable. We expect the interaction term (LRI+)*LC to be significantly different from zero (either positive or negative).

Next, we will identify potential heterogeneous effects between agreements that mention worker rights but do not include strong enforcement or deep cooperation mechanisms, and agreements that mention worker rights and in addition define strong enforcement and/or deep cooperation. We estimate the following equation, which uses agreements without a LC as the reference group:

2 $$ \ln {m}_{ij t}={\alpha}_{it}+{\alpha}_{jt}+{X}_{ij}\gamma +{\beta}^{weak}{LC}_{ij t}^{weak}+{\beta}^{enf}{LC}_{ij t}^{enf}+{\beta}^{coop}{LC}_{ij t}^{coop}+{\beta}^{enf coop}{LC}_{ij t}^{enf coop}+{\varepsilon}_{ij t} $$

with $$ {LC}_{ijt}={LC}_{ijt}^{weak}+{LC}_{ijt}^{enf}+{LC}_{ijt}^{coop}+{LC}_{ijt}^{enf coop} $$

${LC}_{ijt}^{weak}$, ${LC}_{ijt}^{enf}$, ${LC}_{ijt}^{coop}$ and ${LC}_{ijt}^{enfcoop}$ are dummy variables that indicate respectively whether the PTA between countries *i* and* j* in year *t* contains a LC^weak^, LC^enf^, LC^coop^, or LC^enfcoop^. Hence, for instance, ${\beta }^{enf}$ captures the marginal impact of having "only" strong enforcement rules in PTAs compared to not having LCs in PTAs; ${\beta }^{coop}$ the marginal impact of having "only" deep cooperation provisions.

It is important to note that the identification of the $\beta$ coefficients in Eqs. (1) and (2) would ideally be done using bilateral fixed effects instead of our set of bilateral controls ${X}_{ij}$. Actually, this would allow us to identify our effect exclusively within PTAs that move from not having a LC towards having a LC or vice-versa. This is a demanding and convincing identification strategy, but the coefficients will only be identified using the very few agreements where there was a change in the LC over the sample period, i.e. 1995–2014 (only 6 PTAs, see annex 2 Table A2.1), which is too small to put any statistical confidence on the results.^24^ Hence, we use the less demanding identification strategy introduced above that replaces the bilateral fixed effects by the more traditional bilateral trade friction controls, such as distance, contiguity, common colonizer, as well as a measure of the depth of PTAs.

Our empirical strategy contrasts with that of ILO (2016) in several ways. First, they only consider 260 PTAs, whereas our PTA sample contains 437, thereby providing more heterogeneity. Second, they do not control for year-specific importer and exporter fixed effects, and therefore the impact of general labor market reforms or of multilateral resistance terms may be attributed to the LC variable in their study.^25^ Third, the use of an instrumental variable estimator in our paper provides a methodologically more convincing approach to determining the direction of causality.

### Addressing endogeneity

Endogeneity may be a concern because larger trade flows between bilateral trade partners can actually increase the demand for LCs in trade agreements, as they imply higher risks for workers associated with competition from countries with weaker standards. Reverse causality may therefore be biasing the results. There could also be co-founding factors. For example, differences in the degree of frictions in the labor market can be a source of demands for LCs in trade agreements to limit unemployment, but they also can affect the bilateral competitiveness of exporters and import-competing firms, and therefore trade flows (Davis, 1998). Thus, omitted variables can also be biasing the results.

To address these endogeneity concerns, we use an instrumental variable (IV) estimator to explain the inclusion of LCs in PTAs. The predictors we used are borrowed from Raess et al. (2018) and include trade union density among members of the PTAs, whether members have a left-leaning government as measured by Keefer (2012), and the potential labor power (PLP) as measured by Rudra (2002). We first predict the probability that a PTA includes a LC using the values of the member country with the highest degree of union density, left-leaning government, and PLP within each PTA. We then estimate the gravity equation using the predicted probability as an instrument and excluding the observations of the countries with the highest degree of union density, left-leaning government and PLP to satisfy the exclusion restriction. The reason is that union density, left-leaning government, and PLP can affect trade flows through other channels than the presence of a LC. In a standard comparative advantage model, these differences in regulations across member countries are a rationale for trade, which would invalidate the exclusion restriction. By dropping the observations for countries that were used as predictor in the first stage, this is no longer a problem, as, for instance, the value of union density in the member country with the highest union density is unlikely to affect trade flows among the other members of the PTA.^26^

Furthermore, we also implement an alternative IV specific to the North–South PTAs adding in the first-stage equation whether the northern country has a purely majoritarian system (Plurality dummy), borrowed from Postnikov and Bastiaens (2020). Following these authors, we expect only Northern majoritarian systems to be positive and statistically significant on the probability of having LCs in PTAs because “developed countries act as policy-makers while their counterparts act as policy-takers” (Postnikov & Bastiaens, 2020: 355) and majoritarian governments are more likely to be captured by certain interest groups and then more prone to use LCs as a protectionist tool (alternatively, to promote fair trade norms).

Finally, the volume of trade and the choice to implement a PTA with a LC may be jointly determined by the pre-agreement tariff level. We thus re-run all IV estimations adding the pre-agreement MFN tariff, defined at the country-pair level as the average MFN tariff of the importing country weighted by bilateral manufacture trade flows the year before the implementation of the PTA, as a control variable.

## Empirical results

Table 1 reports the results of the estimation of Eq. (1) in a sample of bilateral trade relationships among countries that have a PTA in place. The estimates reported in column (1) suggest that on average LCs do not have a statistically significant impact on bilateral trade among partners belonging to a PTA. All other variables are statistically significant and have the expected sign. In particular, the depth of the PTA has a statistically significant and positive impact on trade flows. As PTAs move beyond market access to include other areas, trade significantly increases. However, when it comes to LCs, there is little support for either the protectionist or the trade-enhancing view.

**Table 1:** Estimation of the LC impact on bilateral manufacture trade flows by subsample, 1995–2014

	(1)	(2)	(3)	(4)	(5)
Variables	ln m_ijt_	ln m_ijt_	ln m_ijt_	ln m_ijt_	ln m_ijt_
**LC**	**-0.062**	**-0.324**	**-0.053**	**-0.208**	0.308**
	*(0.057)*	*(0.214)*	*(0.082)*	*(0.212)*	*(0.146)*
ln (Distance _ij_)	-1.348***	-1.047***	-1.170***	-1.542***	-1.340***
	*(0.029)*	*(0.073)*	*(0.054)*	*(0.091)*	*(0.080)*
Contiguity_ij_	0.160*	0.811***	-0.0796	0.226	0.686*
	*(0.094)*	*(0.152)*	*(0.120)*	*(0.354)*	*(0.356)*
Common Language_ij_	0.734***	0.560***	0.433***	1.083***	0.690***
	*(0.069)*	*(0.147)*	*(0.125)*	*(0.192)*	*(0.111)*
Past Colonial_ij_	0.880***	0.407	0.863***	0.142	1.130***
	*(0.113)*	*(0.433)*	*(0.157)*	*(0.344)*	*(0.178)*
Common Colonial_ij_	0.964***	0.813***	1.365***	0.790***	0.553***
	*(0.099)*	*(0.178)*	*(0.229)*	*(0.287)*	*(0.180)*
Depth of PTA_ijt_	0.491***	0.428***	0.364**	1.416***	1.199***
	*(0.086)*	*(0.163)*	*(0.185)*	*(0.362)*	*(0.201)*
**Sample**	**All**	**South to South**	**North to North**	**North to South**	**South to North**
Observations	62,320	10,914	18,294	8,550	23,372
Adj. R-squared	0.869	0.813	0.908	0.852	0.849
*importer—year Fixed Effects (it)*	*Yes*	*Yes*	*Yes*	*Yes*	*Yes*
*exporter—year Fixed Effects (jt)*	*Yes*	*Yes*	*Yes*	*Yes*	*Yes*

OLS estimates. Standard errors in parentheses are clustered at the dyad level; ^*^p < 0.1; ^**^p < 0.05; ^***^p < 0.01

Source: authors' computation

This is only an average effect across all types of PTAs, which may hide some important heterogeneity. In columns (2) to (5) of Table 1 we show the results for the four sub-samples as described above. While the impact among low-income countries, among high-income countries and from high to low and middle-income countries is not statistically significant, it is statistically significant and positive for exports from low and middle-income countries to high-income countries. This is consistent with hypothesis 1b (and thus contradicts hypothesis 1a). According to the estimates in column (5), the inclusion of a LC in a PTA that includes low and high-income countries is correlated with 36 percent (0.36 = ${\mathrm{e}}^{0.308}-1$) larger bilateral exports from low and middle-income countries to high-income countries, all other things equal. Thus LCs seem to have a strong and positive impact when the exporting country is a low or middle-income country with relatively weaker worker protection and the importing country is a high-income country with relatively stronger worker protection and more concerned firms and consumers.

Next, we more directly test the channel through which LCs are operating by using differences in worker protection across countries as operationalized above. The results are reported in Table 2. The LC variable by itself becomes negative and insignificant, so suggesting again than on average LCs do not have any statistically significant impact on trade flows. However, the interaction of the LC variable with the positive difference in worker protection is positive and statistically significant, suggesting that when the exporter has more violations of workers’ rights than the importer, the impact of the introduction of a LC in a trade agreement is positive. In contrast, when the exporter has less violations of workers’ rights than the importer, the impact is not statistically significant. Again, this suggests that the introduction of a LC has a positive and statistically significant impact on trade where it is expected, i.e., when the exporter has weaker worker protection than the importer. In short, these results not only provide further support for hypothesis 1b, they are also fully consistent with the results from Table 1.

**Table 2:** Impact of LCs on bilateral manufacture trade flows as a function of differences in violations of worker protection between exporters and importers, 1995–2014

	(1)
Variables	ln m_ijt_
**LC**	**-0.071**
	*(0.072)*
**Exporter-Importer LRI + **	**-0.095**
	*(0.076)*
(Exporter-Importer LRI +)*LC	0.068**
	*(0.029)*
**Exporter-Importer LRI-**	**0.016**
	*(0.076)*
(Exporter-Importer LRI-)*LC	**-0.002**
	*(0.034)*
ln (Distance_ij_)	-1.329***
	*(0.029)*
Contiguity_ij_	0.137
	*(0.100)*
Common Language_ij_	0.801***
	*(0.069)*
Past Colonial_ij_	0.813***
	*(0.115)*
Common Colonial_ij_	1.003***
	*(0.100)*
Depth of PTA_ijt_	0.565***
	*(0.088)*
**Sample**	**All**
Observations	57,625
Adj. R-squared	0.873
*importer—year Fixed Effects (it)*	*Yes*
*exporter—year Fixed Effects (jt)*	*Yes*

OLS estimates. Standard errors in parentheses are clustered at the dyad level; ^*^p < 0.1; ^**^p < 0.05; ^***^p < 0.01

Source: authors' computation

**Fig. 4:**
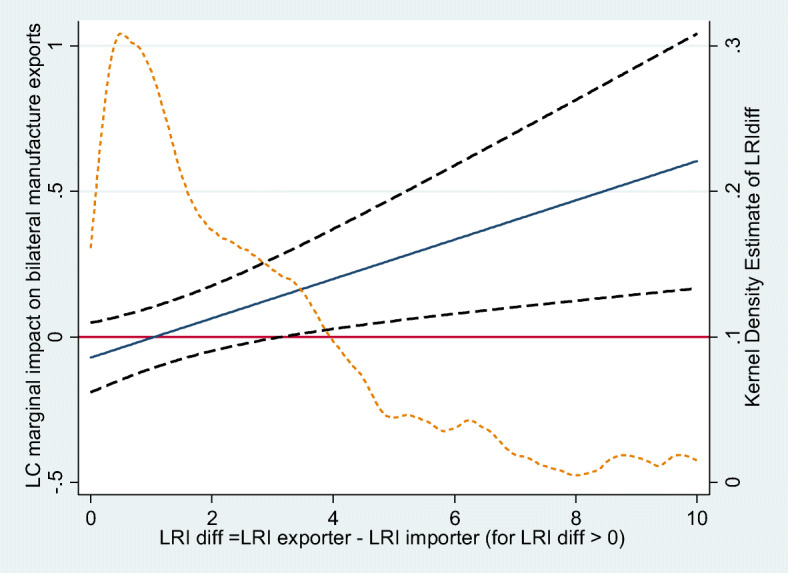
Marginal impact of introducing a LC as a function of difference between exporter and importer violations of workers’ rights. Note: Computed using the estimates reported in Table 2. Source: authors’ computation

Using the estimates reported in Table 2, we compute the marginal impact of introducing a LC on trade flows as a function of the difference between exporter and importer’s worker rights violations, as well as its 95 percent confidence interval. As shown in Fig. 4, the marginal impact of introducing a LC is increasing with the difference in the exporter and importer’s worker rights violations. For a difference in violations of workers’ rights between exporter and importer larger than 3, the impact becomes positive and statistically significant. This suggests that trade can be boosted by the introduction of a LC if the difference between exporter and importer violations of workers’ rights is sufficiently large. Around 20 percent of the trading relationships between exporter and importers involve a difference in worker rights larger than 3. This includes exports from Algeria to France, or Malaysia to Japan, Peru towards Canada, where the difference in violations of worker rights is around 4, and the increase in exports associated with the inclusion of a LC is estimated around 20 percent. In the case of exports from Nigeria to Sweden where the differences in workers’ right is even larger (around 6), the increase is as large as 34 percent.

A further source of heterogeneity is likely to be related to differences in LCs themselves. In the results reported in Table 1, we consider that the impact on trade flows of simply mentioning worker protection as an objective in the preamble is equivalent to a LC with fines for violating worker protection. This is a strong assumption that we relax in Table 3. As described in Fig. 3, most of the variation in PTAs with labor-related strong enforcement and/or deep cooperation mechanisms occurs in the sample of agreements between low and high-income countries, which is where we expect the impact of LCs to be the strongest regardless of the direction of the impact. Thus, we only focus on this sub-sample in Table 3.

**Table 3:** Estimation of the impact of North–South LCs with enforcement and cooperation on South-North manufacture trade flows, 1995–2014

	(1)	(2)
VARIABLES	ln m_ijt_	ln m_ijt_
**LC**	0.308**	
	*(0.146)*	
**LC**^**weak**^** wo strong enforcement or deep cooperation**	**-**	0.244**
		*(0.076)*
**LC**^**coop**^** with deep cooperation "only"**	**-**	1.315***
		*(0.149)*
**LC**^**enf**^** with strong enforcement "only"**	-	**-0.313**
		*(0.209)*
**LC**^**enfcoop**^** with strong enforcement and deep cooperation**	-	**-0.190**
		*(0.137)*
ln (Distance_ij_)	-1.340***	-1.334***
	*(0.080)*	*(0.031)*
Contiguity_ij_	0.686*	0.664***
	*(0.356)*	*(0.104)*
Common Language_ij_	0.690***	0.653***
	*(0.111)*	*(0.039)*
Past Colonial_ij_	1.130***	1.147***
	*(0.178)*	*(0.051)*
Common Colonial_ij_	0.553***	0.489***
	*(0.180)*	*(0.066)*
Depth of PTA_ijt_	1.199***	0.887***
	*(0.201)*	*(0.097)*
**Sample**	**South to North**	
Observations	23,372	23,372
Adj. R-squared	0.849	0.850
*importer—year Fixed Effects (it)*	*Yes*	*Yes*
*exporter—year Fixed Effects (jt)*	*Yes*	*Yes*

OLS estimates. Standard errors in parentheses are clustered at the dyad level; ^*^p < 0.1; ^**^p < 0.05; ^***^p < 0.01. The reference group is “PTAs with no LCs”. We construct the four variables capturing the four types of LCs as being mutually exclusive, i.e., ${LC}_{ijt}={LC}_{ijt}^{weak}+{LC}_{ijt}^{enf}+{LC}_{ijt}^{coop}+{LC}_{ijt}^{enfcoop}$

Source: authors' computation

The estimates reported in the second column of Table 3 suggest that the positive impact of LCs on South-North trade flows is mostly explained by institutionalized cooperation provisions in the LC. Enforcement mechanisms do not seem to have much of an impact, whether or not they are associated to deep cooperation measures (coefficients are not significantly different from zero). This is inconsistent with hypothesis 2a. Most of the impact measured in the first column, which simply reproduces the main result of Table 1, is due to the impact of LCs with only deep cooperation mechanisms. This provides strong support for hypothesis 2b.

Table 4 reports the results of the second-stage of the two-stage least squares estimator. They confirm the findings of Table 1 where the LC has a significant and positive impact in North–South PTAs and for trade flows from South to North only. This is robust in both columns (5) and (6). Column (5) uses in the first-stage estimation the three instruments evidenced in Raess et al. (2018) – as in columns (1)-(4). Column (6) adds in its first stage a dummy if the Northern country is majoritarian representation following Postnikov and Bastiaens (2020).^27^ According to the Cragg-Donald Wald F statistic we can reject the null hypothesis of weak instrument.^28^

**Table 4:** IV Estimation of the LC impact on bilateral manufacture trade flows by subsample, 1995–2014

	(1)	(2)	(3)	(4)	(5)	(6)
Variables	ln m_ijt_	ln m_ijt_	ln m_ijt_	ln m_ijt_	ln m_ijt_	ln m_ijt_
**LC**	**-0.282**	**0.144**	**0.266**	**-1.043**	1.087**	0.957*
	*(0.236)*	*(1.866)*	*(2.935)*	*(1.385)*	*(0.496)*	*(0.525)*
ln (Distance_ij_)	-1.311***	-0.972***	-1.106***	-1.732***	-1.443***	-1.434***
	*(0.043)*	*(0.138)*	*(0.177)*	*(0.145)*	*(0.098)*	*(0.099)*
Contiguity_ij_	0.026	1.367***	-0.0971	0.615	0.704	0.730
	*(0.133)*	*(0.261)*	*(0.191)*	*(0.540)*	*(0.573)*	*(0.576)*
Common Language_ij_	0.576***	0.213	0.319	0.963***	0.615***	0.619***
	*(0.088)*	*(0.366)*	*(0.238)*	*(0.240)*	*(0.119)*	*(0.120)*
Past Colonial_ij_	1.046***	0.506	0.959***	0.133	1.155***	1.153***
	*(0.121)*	*(0.828)*	*(0.176)*	*(0.380)*	*(0.177)*	*(0.178)*
Common Colonial_ij_	0.986***	1.046**	1.448*	0.440	0.443**	0.449**
	*(0.133)*	*(0.442)*	*(0.773)*	*(0.365)*	*(0.208)*	*(0.208)*
Depth of PTA_ijt_	0.403***	0.566	0.403	1.355*	1.225***	1.207***
	*(0.139)*	*(0.445)*	*(0.300)*	*(0.700)*	*(0.335)*	*(0.342)*
**Sample**	**All**	**South to South**	**North to North**	**North to South**	**South to North**	**South to North**
Observations	48,008	3,572	15,679	6,474	21,297	21,297
Cragg-Donald Wald F stat ^a/^	4,423	47	21	317	6,083	3,430
*importer—year Fixed Effects (it)*	*Yes*	*Yes*	*Yes*	*Yes*	*Yes*	*Yes*
*exporter—year Fixed Effects (jt)*	*Yes*	*Yes*	*Yes*	*Yes*	*Yes*	*Yes*

IV estimates. Standard errors in parentheses are clustered at the dyad level; *p < 0.1; **p < 0.05; ***p < 0.01. First-stage estimations are reported in Table A3

a/ The Stock and Yogo (2005) weak identification test critical value at 10% maximal IV size (for Cragg-Donald F statistic and i.i.d. errors) is 16.38

Source: authors' computation

Finally, as reported in annex 4 Table A4, the pre-agreement tariff is not significantly different from zero except on the whole sample (column 1) and our results on the LC impact are robust to the inclusion of this variable in the gravity equation: LC has a significant (and positive) impact only in the South to North sample (columns 5 and 6).

## Discussion

What might explain the (positive) impact of institutionalized cooperation LCs but not of strong enforcement LCs? First, Northern countries using strongly enforceable LCs as a protectionist tool to withdraw preferential market access of developing countries in the context of PTAs never actually happens. With one exception (US-Guatemala case under the CAFTA-DR PTA), states have so far been reluctant to launch formal adjudication procedures to settle their trade-related labor disputes. Major players have tended to shy away from using dispute settlement provisions. The EU in particular has shown little appetite for pressuring partners found in violation of their labor commitment, although this is changing as of late. The EU Commission’s 15 points action plan launched in February 2018 with the aim to step up efforts for the effective implementation of its trade and sustainability chapters foresees more assertive enforcement.^29^ In December of that same year, the EU Commission announced it had requested formal consultations with the Korean government regarding the implementation of the labor commitments under the EU-Korea PTA.^30^ In any case, until most recently the political will to use available tools under dispute settlement has been wanting and the only case going for full-scale arbitration (US-Guatemala) has been lost by the US to the surprise of many after a lengthy and cumbersome procedure, which does not bode well for future labor law complaints under arbitration mechanisms of a PTA unless the mechanisms are strengthened (Paiement, 2018). In short, protectionist interests and motives in the North as a possible driving force behind a negative relationship between enforceable LCs and bilateral trade seems to have no substantive basis.

Second, the kind of deep cooperation described in some PTAs does actually happen. Among the key promoters of labor provisions in PTAs, Canada, Chile, the EFTA, the EU, New Zealand and the US have all established institutional mechanisms for third party involvement (ILO, 2016: Table 3.1). For instance, NAFTA pioneered the participation of non-state actors in the implementation phase as it introduced elements (reporting, dialogue) that were reinforced (involvement in cooperation, advising governments via recommendations) in subsequent agreements (ILO, 2016: Box 3.1). Also, "new generation" EU PTAs, starting with EU-Korea (2010), provide for highly institutionalized civil society involvement mechanisms, including domestic advisory groups (DAGs) that comprise, but are not limited to, representatives of the social partners, and a Civil Society Dialogue Forum where civil society organizations of the trading parties meet (Orbie et al., 2018). Systematic information on the frequency of meetings of these bodies is difficult to come by. A glance at the EU suggests that they can be frequent. Under the EU-Korea PTA, 19 meetings of the EU DAG and 6 meetings of the EU-Korean Civil Society Forum had taken place by the end of 2019,^31^ while similar civil society meetings are occurring in the EU PTAs with Central America (2012), Peru-Colombia (2012), and Moldova (2014), amongst others (Orbie et al., 2018). Clearly, those bodies do not just exist on paper, but officials do actually meet. And they do oversee cooperation activities on the ground.

Indeed, one of the most extensive labor capacity building program has been rolled out in the context of the CAFTA-DR agreement with the American government spending over USD142 million over the period 2005–2010 to promote freedom of association and social dialogue.^32^ The US has carried out capacity building programs focusing amongst others on the strengthening of the labor inspection systems under the US-Peru (2006), US-Panama (2007), US-Colombia (2006) PTAs and, in collaboration with the ILO, in the US-Jordan (2000), US-Bahrein (2004) and US-Oman (2006) PTAs (Dewan & Ronconi, 2018: Table 1). Similarly, in the PTA context, Canada has implemented a CAD 2.5 million technical assistance program strengthening Costa Rican labor administration, and rolled out a six-month training course for Peruvian labor inspectors and a project aimed at promoting social dialogue (ILO, 2013: 82–3). Also, some secondary players have deployed cooperative activities. For instance, New Zealand implemented technical cooperation activities vis-à-vis Thailand over the period 2006–2009, including capacity building related to occupational health and safety and labor inspection, and a long-term project on the mediation of labor disputes (ILO, 2013: Table 3.6). Chile, classified as a Northern country in our analysis, is another case in point. According to the Labor Attaché with the Permanent Mission of Chile to the UN Pablo Lazo Grandi (ILO, 2017: 53), "Chile’s experience in the implementation of labor provisions can be characterized as active in terms of political and social dialogue (including governments and/or social partners), based on the development of cooperative activities, and with no activation of dispute resolution mechanisms". Examples of Chilean PTAs with such implementation activities include amongst others Canada-Chile (1996), US-Chile (2003), the Trans-Pacific Strategic Economic Partnership Agreement (2005), and Chile-Peru (2006).

Related, and third, to our knowledge there is no robust empirical evidence suggesting that strongly enforceable LCs improve labor standards ex post while there is such evidence regarding institutionalized cooperation LCs (Postnikov & Bastiaens, 2014; Raess & Sari, 2020b). Also, to the extent that LCs with deep cooperation mechanisms lead to labor upgrading in developing countries while enforceable LCs do not, the former provides a more credible signal regarding adequate labor standards, which in turn should increase product demand.

This begs the question, to whom do LCs with deep cooperation send a credible signal? There are good reasons to doubt that concerned consumers know who are their country’s PTA partners and which PTAs have strong LCs and which have not. The literature on foreign economic policy preference formation has shown that individuals often have great difficulty in understanding their own egocentric policy preferences or are simply ignorant (Hainmueller & Hiscox, 2006; Mansfield & Mutz, 2013; Rho & Tomz, 2017). If they do not know what is in their best material self-interest, it is unlikely that trading relationships and design features of PTAs will inform their purchasing decisions.

Accordingly, to the extent that the positive impact of (institutionalized cooperation) LCs on Southern exports to the North runs via the demand-side mechanism, concerned firms in the North are likely to play a more important role than consumers in driving demand for fair trade products. In the context of private regulation of standards in global value chains, Distelhorst and Locke (2018) demonstrate the key role played by concerned firms in the North by providing evidence for a firm-level mechanism linking exporter compliance with social standards and increased orders by importers. Our results appear to suggest that this mechanism plays out at the macro-level as well, with exporters in countries that sign PTAs with strong LCs being more likely to both improve labor standards and establish relationships with risk adverse importers (and thereby increase trade flows).

In sum, labor-related institutionalized cooperation is the channel through which increased demand by Northern firms operates because LCs with deep cooperation mechanisms are about providing information and finding solutions to violations of labor rights. Through the participation of employer representatives (and trade unions) in the monitoring and implementation phases, firms indirectly have access to information about improvements in the law and in practice, which can inform their purchasing decisions. And the involvement of the social partners and other third parties yields the promise of stronger improvements in labor standards, because they have a say at the table when country-level priorities and action plans are decided and because they participate in their implementation on the ground, providing “teeth” to capacity building activities.

Still, strong LC enforceability through sanction measures might pave the way for institutional capture by lobby groups for protectionist purposes, which might prove right those who argue that LCs amount to hidden protectionism. When sanctions are present, it would seem that protectionism wins over the other mechanisms or the two effects cancel each other out. This is, in any case, what our results suggest, even though they are statistically insignificant (see Table 3).

## Conclusion

Some argue that LCs in PTAs are hidden protectionist tools that hurt exports of low-income countries with weaker labor standards due to either cost increases in low-income countries that need to satisfy these LCs to benefit from improved market access or the use of enforceable LCs as a protectionist device in high-income countries. Others have argued that the introduction of LCs can instead help exporters in low-income countries become more productive or signal adequate levels of worker protection to concerned stakeholders in countries with stronger labor standards, which will increase demand from final consumers and firms involved in global supply chains.

To assess which of these views predominates, we use the gravity model of international trade to assess the impact of the introduction of (various types of) LCs in PTAs on bilateral trade flows. We found that on average, across all types of PTAs, there is no statistically significant impact of the introduction of LCs on bilateral trade flows. However, these average results hide interesting heterogeneity. The impact of LCs is statistically significant, large and positive on exports of low and middle-income countries towards high-income countries. Consistent with this result, in a direct test of the channel through which LCs are operating, we found that the inclusion of a LC has a positive impact on trade flows when the exporter has weaker labor standards then the importer. Moreover, the impact of LCs in North–South PTAs is highly significant when LCs are accompanied by institutionalized cooperation. In contrast, LCs with strong enforcement mechanisms, whether accompanied by institutionalized cooperation or not, do not have a statistically significant impact on bilateral trade flows in North–South PTAs.

To sum up, the impact of LCs is strong where they are expected to have an impact, and it is mainly driven by institutionalized cooperation provisions in the LCs. Contrary to what is sometimes suggested, low-income countries with weaker labor standards should not fear the introduction of LCs as a protectionist tool in PTAs as they help rather than hinder their market access to high-income countries. Both low and high-income countries should embrace LCs with institutionalized cooperation mechanisms since the greater trade they generate is at the same time associated with improved labor standards in low-income countries (Raess & Sari, 2020b). As such, they meet the concerns of two core constituencies in high-income countries, the fair traders, by improving labor standards abroad, and those who seek protection, by leveling the playing field for workers and businesses at home, and thereby they help to legitimize the policy of free trade.

## Supplementary Information

Below is the link to the electronic supplementary material.

ESM 1 Supplementary file1 (PDF 368 KB)

## Footnotes

1 The one existing study is ILO (2016), which we discuss below.

2 In 1984, a labor rights clause was introduced for the first time in the GSP (Compa & Vogt, 2001). However, it is important to note that preferences are unilaterally given in the GSP, and therefore the determinants and impact of GSP programs follow a different logic. Focusing on the US GSP, Hafner-Burton et al. (2019) find that rights-based considerations account for the maintenance of country-level trade benefits irrespective of import pressure while protectionist concerns drive suspensions from GSP eligibility at the product-level.

3 This excludes PTAs that simultaneously follow a goal of political integration, such as EC Treaties or the Andean Community (1988). The preamble of the Global System of Trade Preferences among Developing Countries (GSTP), a PTA signed at Belgrade in 1988, specifies an objective of creation of employment, but it is debatable whether such reference counts as a labor provision (Raess & Sari, 2020a).

4 This is excluding the Trans-Pacific Partnership.

5 Freedom of association and the right to collective bargaining; the elimination of forced and compulsory labor; the abolition of child labor; and the elimination of discrimination in the workplace.

6 Lechner (2018) examines the impact of environmental and labor standards in US PTAs on US outward foreign direct investment. Berger et al. (2020) investigate the trade effects of environmental provisions in PTAs.

7 The 1999 U.S.-Cambodia Bilateral Textile Trade Agreement linked preferential market access to labour standards compliance. The ILO’s Better Factories Cambodia program monitors working conditions in Cambodian garment factories and assesses conditions relative to ILO Core Labour Standards and Cambodian labour law. See http://betterfactories.org/.

8 The rise of shareholder activism or ethically focused investment funds are also evidence of increased demand for labor standards.

9 A follow-up study finds mixed results: studying a reputation-conscious North American retailer, Amengual et al. (2020) find that orders from suppliers with poor labor compliance were more likely to be cancelled while they did not increase when labor standards improved.

10 For a detailed discussion of the role and effectiveness of LCs with institutionalized cooperation mechanisms in addressing poor labor standards, see Raess and Sari (2020b).

11 Dewan and Ronconi (2018) find that LCs in US PTAs promote better labor law enforcement in Latin American partner countries, but the effect crucially depends on political will and pressure as well as resources devoted to capacity building – suggesting that the combination of the two approaches is most effective.

12 For a detailed assessment of the strengths and limitations of those datasets with respect to the mapping of labor provisions, see Raess and Sari (2018).

13 Accordingly, a PTA is coded as having no LC if the only provision is a reference to "create employment opportunities" in the preamble as there is no recognized right to employment.

14 Fourteen agreements are not included as they focus on services (e.g., EFTA services) or specific products (e.g., Andean sugar protocol); six have been signed in 2015 so they are out of sample; others correspond to amendments or revisions of existing agreements (with only a potential evolution in their LCs – see explanation in annex 2).

15 We define "Northern" countries as "High-income countries" (OECD and non OECD) in the July 2017 World Bank classification, "Southern" countries as the "Middle and Low-income countries".

16 Note that this implies that our estimated impact of LCs on bilateral trade flows does not include the impact of LCs on labor laws at the national level that would affect all sectors and bilateral exports homogenously.

17 As reported in Table A1 of annex 1, agreements with LCs have a significantly higher depth index.

18 Except for the measure of PTA depth, all these data are from BACI database provided by the CEPII. See annex 1 for details on variable definitions.

19 We have less than 6% of zeros in our sample of country pairs that have PTAs in place (while in more general setups samples usually include around 43% of zeros), so we do not consider zero trade flows. However, as a robustness check, we perform a Pseudo-Poisson Maximum Likelihood on the benchmark estimations and sample including zero trade flows (Table 1) and find qualitatively same results.

20 One may be tempted to explore the heterogeneity of the impact across sectors, and for example see whether more labor intensive sectors are likely to be more affected by the inclusion of LCs. The implicit assumption here is that technology within sectors is the same across countries. This however is counterfactual as agriculture or mining are often more capital intensive than manufacture in rich countries whereas the opposite is true in poor countries.

21 The type of relationships is defined at the bilateral level and not at the PTA level. For example, even if NAFTA is coded “North-South”, only the dyads “Mex-US” and “Mex-Can” are defined as export flows from low and middle-income to high-income countries (and included in the relevant sample), the trade between US and Canada is taken into account in the sample of high-income countries.

22 Note that this is consistent with exports from the South to the North as our data suggests that a) worker protection is stronger in high-income countries, and b) differences in worker protection are larger when looking at dyads belonging to different income groups.

23 As in Kucera and Sari (2019: footnote no. 15), we drop fourteen countries from the sample “on the grounds that their implausibly favorable scores primarily reflected a large degree of under-reporting of violations [...]. These countries are Afghanistan, Angola, Azerbaijan, Chad, Congo, Gabon, Gambia, Guinea, Kyrgyzstan, Maldives, Somalia, South Sudan, Tajikistan and Yemen.” Note that results are qualitatively identical if these countries are included in our sample.

24 Table A2.2 in annex 2 shows the results for this specification.

25 One may argue that the inclusion of year-specific exporter fixed effects may be partly capturing the impact that LCs may have on trade flows if the change in the enforcement of laws homogenously affects all sectors and therefore all bilateral trade relationships. While this is unlikely, we need to recognize that the inclusion of these fixed effects partly captures the impact of LCs on trade flows.

26 Note, however, that the results remain qualitatively the same without dropping the observations for countries that were used as instrument in the first stage.

27 The results of the first-stage estimation are provided in Table A3 in annex 3.

28 Note that our second-stage equation is exactly identified as we use only one instrument: the predicted value of the probability that a PTA includes a LC (estimated in the first-stage equation provided in annex 3). So we cannot perform tests that require overidentification like the Hansen J statistic.

29 http://trade.ec.europa.eu/doclib/press/index.cfm?id=1803 (accessed 25 April 2020).

30 See http://trade.ec.europa.eu/doclib/press/index.cfm?id=1961 (accessed 25 April 2020). Note that labor provisions under the EU-Korea PTA are not enforceable through sanctions.

31 https://www.eesc.europa.eu/en/sections-other-bodies/other/republic-korea-domestic-advisory-group-under-fta/events (accessed 25 April 2020).

32 https://ustr.gov/about-us/policy-offices/press-office/fact-sheets/2011/may/cafta-dr-labor-capacity-building (accessed 3 November 2020).
