# Supplementary material for: Labor clauses in trade agreements: Hidden protectionism?
Source: Rev Int Organ. 2021 May 29;17(3):453–83. doi: 10.1007/s11558-021-09423-3 (PMC9212734; doi:10.1007/s11558-021-09423-3)
Supplement: Supplementary file 1 — Supplementary file1 (PDF 368 KB) [file 11558_2021_9423_MOESM1_ESM.pdf]

Online Appendix

Labor Clauses in Trade Agreements: Hidden Protectionism?

Annex 1. Control variables

The United Nations Statistical Division (COMTRADE database) provides the original trade data from customs declarations. CEPII uses COMTRADE to create BACI that reconciles differences in the declarations between exporters and importers.[1] This harmonization procedure enables to extend considerably the number of countries for which trade data are available, as compared to the original COMTRADE dataset, but only from 1995 onwards. m_{ijt} is the value of imports of country i from country j in year t (in thousands of US dollars).

The geodesic distance is calculated following the great circle formula, which uses latitudes and longitudes of the most important cities/agglomerations (in terms of population); see Mayer and Zignago (2011) for more details. Other gravity variables include dummy variables indicating whether the two countries are contiguous, share a common language, have had a common colonizer after 1945 or have ever had a colonial link. The common language dummy is set to one if a language is spoken by at least 9 percent of the population in both countries. Trying to give a precise definition of a colonial relationship is obviously a difficult task. Colonization is here a fairly general term that we use to describe a relationship between two countries, independently of their level of development, in which one has governed the other over a long period of time and contributed to the current state of its institutions. All the gravity variables are from CEPII's BACI database.

[1] See http://www.cepii.fr/CEPII/en/bdd_modele/presentation.asp?id=37.

The measure of depth is a dummy variable based on an additive index that combines seven key provisions that can be included in PTAs (see Dür, Baccini and Elsig 2014). The first provision captures whether the agreement foresees that all tariffs (with limited exceptions) should be reduced to zero (that is, whether the aim is to create a full free trade area). The other six provisions capture cooperation that goes beyond tariff reductions, in areas such as services trade, investments, standards, public procurement, competition and intellectual property rights. For each of these areas, Dür, Baccini and Elsig (2014) code whether the agreement contains any substantive provisions. We use an updated variable of their measure that was kindly provided to us by Andreas Dür. The dummy we use equals unity if the depth index is larger or equal to its median, namely 3. As shown in table below, PTAs with LCs are significantly "deeper" than PTAs without LCs. "Depth_index" refers to the index (in the range of 0-7) of Dür, Baccini and Elsig (2014) and "depth_dummy" refers to our dummy.

In annex 4 we use as additional control variable the pre-agreement tariff. More precisely, the pre-agreement MFN tariff is the latest MFN tariff available for a country before the years before the implementation of the PTA (from t-1 to t-5). We extract from the World Integrated Trade Solution (WITS - UNCTAD TRAINS database) the average MFN tariff only for on manufacture sectors, weighted by bilateral manufacture trade flows.

In fine, we use a sample of 64,297 observations, including 5,605 country-pairs and 437 PTAs over 1995-2014 (PTAs are reported in annex 4 Table A4).

Table A1. Some descriptive statistics

	Variable	Obs	Mean	Std. Dev.	Min	Max
LC=0	depth_index	268	**2.21**	1.82	0	7
LC=1	depth_index	169	**3.87**	2.06	0	7

Test		diff	Std. Err.	[95% Conf.	Interval]
	LC 0 vs 1	-1.67	.19	-2.03	-1.30

	Variable	Obs	Mean	Std. Dev.	Min	Max
LC=0	depth_dummy	268	**.59**	.49	0	1
LC=1	depth_dummy	169	**.93**	.25	0	1
Test			Contrast	Std. Err.	[95% Conf.	Interval]
	LC 0 vs 1		-0.35	.04	-0.43	-0.27

Source: authors' computation.

Annex 2. Some assumptions to compute LCs

First, we only have the date of signature, thus we consider all agreements signed since 1990 (first year of the LABPTA dataset; for details, see Raess & Sari, 2018) until 2014 (latest year of available trade data).

Second, if an agreement is amended without any changes in its LC, this is not taken into account (only the first date is used to define the dummy); if an agreement is amended with a change in its LCs, this is taken into account. For instance, if a subset of countries signs a first agreement with no LC and, several years after, the same subset of countries sign a new agreement with LC, we consider that the first agreement is amended, the LC dummy is set to unity for all country pairs within this PTA.

Evolution in LCs over 1995-2014 within a subset of countries is important in our study as this is the way the LCs impact is identified in the gravity equation with dyad fixed effects. Over the 437 PTAs considered in this study, only 9 reports action in their LCs, and only 6 over 1995-2014.

Table A2.1. Evolution in LCs over 1990-2014 within a PTAs

PTA	no LC	LC	Enforc.	Deep coop.
Chile Mexico	1991	1998	No	No
Chile Colombia	1993	2006	No	Yes
EC Slovenia Europe Agreement	1993	1996	Yes	No
Colombia Panama	1993	2013	No	Yes
Chile Ecuador	1994	2008	No	No
Chile Peru	1998	2006	No	Yes
EC Estonia Europe Agreement	*1994*	*1995*	*Yes*	*No*
EC Latvia Europe Agreement	*1994*	*1995*	*Yes*	*No*
EC Lithuania Europe Agreement	*1994*	*1995*	*Yes*	*No*

Note: PTAs in italic reports action in their LCs that are out of the trade sample (1995-2014).
Source: authors' computation.

Table A2.2. Estimation of the impact of LC with enforcement and cooperation on bilateral manufacture trade flows, 1995-2014

	(1)	(2)
VARIABLES	ln m_{ijt}	ln m_{ijt}
**LC**	**-0.009**	**-0.036**
	(0.087)	*(0.095)*
**LC^{enf} with strong enforcement**		**0.032**
		(0.115)
**LC^{coop} with deep cooperation**		**0.014**
		(0.109)
Observations	64,297	64,297
Adj. R-squared	0.949	0.949
importer - exporter Fixed Effects (ij)	*Yes*	*Yes*
importer - year Fixed Effects (it)	*Yes*	*Yes*
exporter - year Fixed Effects (jt)	*Yes*	*Yes*

Note: OLS estimates. Standard errors in parentheses are clustered at the dyad level; $^{*}p<0.1$; $^{**}p<0.05$; $^{***}p<0.01$.
Source: authors' computation.

Annex 3. First stage results of two-stage least square estimation

Table A3. First-stage logit estimation, at the PTA level

	(1)	(2)	(3)	(4)	(5)	(6)
VARIABLES	LC	LC	LC	LC	LC	LC
PLP	-0.153**	-0.293	-0.355**	-0.288**	-0.304**	-0.292**
	(0.073)	*(0.388)*	*(0.181)*	*(0.116)*	*(0.118)*	*(0.138)*
Left government	0.228	1.211*	-0.459	0.190	0.085	0.066
	(0.257)	*(0.701)*	*(0.493)*	*(0.452)*	*(0.439)*	*(0.433)*
Union density	0.030***	0.035***	0.014	0.032**	0.031**	0.032**
	(0.008)	*(0.013)*	*(0.013)*	*(0.014)*	*(0.014)*	*(0.014)*
Plurality dummy					-	0.097
						(0.549)
**Sample**	**All**	**South to South**	**North to North**	**North to South**	**South to North**	**South to North**
Observations	324	97	91	135	136	136

*Note: Logistic regression estimates. Robust standard errors in parentheses; *p<0.1; **p<0.05; ***p<0.01.*
Source: authors' computation.

Annex 4. Robustness to the introduction of the pre-agreement tariff variable

Table A4. IV Estimation of the LC impact on bilateral manufacture trade flows with introduction of the pre-agreement tariff - by subsample, 1995-2014

VARIABLES	(1)<br>ln m_{ijt}	(2)<br>ln m_{ijt}	(3)<br>ln m_{ijt}	(4)<br>ln m_{ijt}	(5)<br>ln m_{ijt}	(6)<br>ln m_{ijt}
**LC**	**-0.282**	**-0.539**	**1.351**	**-0.933**	**1.094****	**0.957***
	(0.236)	*(2.089)*	*(8.175)*	*(1.338)*	*(0.500)*	*(0.530)*
ln ($Distance_{ij}$)	-1.310***	-1.044***	-1.136***	-1.733***	-1.471***	-1.460***
	(0.043)	*(0.127)*	*(0.427)*	*(0.144)*	*(0.102)*	*(0.102)*
$Contiguity_{ij}$	-0.011	1.231***	-0.029	0.818	0.685	0.711
	(0.132)	*(0.256)*	*(0.354)*	*(0.538)*	*(0.574)*	*(0.578)*
Common $Language_{ij}$	0.578***	0.072	0.263	0.954***	0.609***	0.613***
	(0.089)	*(0.399)*	*(0.516)*	*(0.242)*	*(0.120)*	*(0.120)*
Past $Colonial_{ij}$	1.042***	0.285	0.934***	0.124	1.155***	1.152***
	(0.121)	*(0.894)*	*(0.226)*	*(0.388)*	*(0.177)*	*(0.178)*
Common $Colonial_{ij}$	1.008***	0.996**	1.822	0.481	0.417**	0.424**
	(0.136)	*(0.482)*	*(2.279)*	*(0.383)*	*(0.213)*	*(0.213)*
Depth of PTA_{ijt}	0.419***	0.635	0.470	1.315*	1.247***	1.228***
	(0.139)	*(0.489)*	*(0.666)*	*(0.691)*	*(0.342)*	*(0.349)*
Pre-agreement $Tariff_{ij}$	1.136**	0.965	-1.148	0.577	-0.076	-0.051
	(0.444)	*(1.594)*	*(7.288)*	*(0.630)*	*(1.102)*	*(1.101)*
**Sample**	**All**	**South to South**	**North to North**	**North to South**	**South to North**	**South to North**
Observations	46,751	3,330	15,133	6,370	20,958	20,958
importer - year Fixed Effects (it)	*Yes*	*Yes*	*Yes*	*Yes*	*Yes*	*Yes*
exporter - year Fixed Effects (jt)	*Yes*	*Yes*	*Yes*	*Yes*	*Yes*	*Yes*

*Note: IV estimates. Standard errors in parentheses are clustered at the dyad level; *p<0.1; **p<0.05; ***p<0.01.*
Source: authors' computation.
